# Rheological Properties of Silica-Fume-Modified Bioasphalt and Road Performance of Mixtures

**DOI:** 10.3390/ma17092090

**Published:** 2024-04-29

**Authors:** Gui Hou, Yanhua Xue, Zhe Li, Weiwei Lu

**Affiliations:** 1 Institute of Cold Region Science and Engineering, Northeast Forestry University, Harbin 150040, China; nmgjky2024@sina.com; 2 Northeast Forestry University Engineering Consulting and Design Research Institute, Ltd., Harbin 150040, China; 3 Inner Mongolia Autonomous Region Transportation Science and Development Research Institute, Hohhot 010051, China; nmgjtysbwh@163.com; 4 National Key Laboratory of Green and Long-Life Road Engineering in Extreme Environment (Changsha), Changsha University of Science & Technology, Changsha 410114, China; lww_cs@csust.edu.cn; 5 National Engineering Research Center of Highway Maintenance Technology, Changsha University of Science & Technology, Changsha 410114, China

**Keywords:** bio-oil, silica fume, rheological properties, bioasphalt

## Abstract

The objective of this research is to enhance the high-temperature antirutting and antiaging characteristics of bioasphalt. In this study, silica fume (SF) was selected to modify bioasphalt. The dosage of bio-oil in bioasphalt was 5%, and the dosage of SF was 2%, 4%, 6%, 8%, and 10% of bioasphalt. The high- and low-temperature characteristics, aging resistance, and temperature sensitivity of Bio + SF were evaluated by temperature sweep (TS), the multiple stress creep recovery (MSCR) test, the bending beam rheology (BBR) test, and the viscosity test. Meanwhile, the road behavior of the Bio + SF mixture was evaluated using the rutting test, low-temperature bending beam test, freeze–thaw splitting test, and fatigue test. The experimental results showed that the dosage of SF could enhance the high-temperature rutting resistance, aging resistance, and temperature stability of bioasphalt. The higher the dosage of SF, the more significant the enhancement effect. However, incorporating SF weakened bioasphalt’s low-temperature cracking resistance properties. When the SF dosage was less than 8%, the low-temperature cracking resistance of Bio + SF was still superior to that of matrix asphalt. Compared with matrix asphalt mixtures, the dynamic stability, destructive strain, freeze–thaw splitting strength ratio, and fatigue life of 5%Bio + 8%SF mixtures increased by 38.4%, 49.1%, 5.9%, and 68.9%, respectively. This study demonstrates that the development of SF-modified bioasphalt could meet the technical requirements of highway engineering. Using SF and bio-oil could decrease the consumption of natural resources and positively reduce environmental pollution.

## 1. Introduction

Petroleum asphalt is a nonrenewable resource. However, with the increasing cost of energy and the strong global demand for petroleum resources, it is an inevitable trend to find new binders to modify or replace petroleum asphalt [1]. Amar K. Mohanty, in his study, pointed out that biomass energy sources have attracted the attention of many researchers for their environmental friendliness, wide sources, large storage capacity, low price, and sustainability [2]. Biomass is mainly derived from crop residues, waste oils and fats, plants, animal feces, municipal waste, wood chips, etc. Biomass energy sources can be converted through a series of transformations and eventually become a new green road material with properties similar to those of petroleum asphalt, i.e., bioasphalt [3]. Applying bioasphalt produced from these biomass materials to the road field could enhance the characteristics of asphalt, deal with waste, protect the environment, and develop a sustainable economy [4].

Julian Mills-Beale et al. [5] utilized a swine waste biobinder to prepare bioasphalt and investigated its rheological characteristics and modification mechanism. It was found that pig waste is compatible with asphalt and effectively improves the low-temperature characteristics. However, it reduced the aging resistance and high-temperature stabilization. Lei et al. [6] researched the effect of varying oils on the low-temperature behavior of asphalt. The study findings exhibited that the oil can effectively strengthen the low-temperature characteristics, and the asphalt mixtures have superior low-temperature anticracking characteristics. Sun et al. [7] researched waste-cooking-oil-modified asphalt’s mechanical, chemical, and rheological characteristics. Silvia Caro et al. [8] researched the chemical, rheological, and thermodynamic characteristics of bagasse, corn kernel, and rice husk bio-oils on bitumen. Yang et al. [9] used wood in the form of woodchips, sawdust, and shavings to produce bio-oils and investigated the effects on the elemental composition of the asphalt, chemical composition, oxidative aging, and compatibility. Several studies showed that most bioasphalts prepared from various biomass resources could enhance the low-temperature characteristics and degrade mixtures’ optimal mixing and compaction temperatures. However, various bio-oils can adversely affect petroleum asphalt’s antiaging and high-temperature characteristics [10]. The poor bioasphalt performance at high temperatures restricts biomass asphalt’s wide-scale utilization in pavement construction.

Therefore, researchers have compensated for its performance by adding modifiers to biomass asphalt to enhance its high-temperature stabilization and antiaging characteristics. Sara R.M. Fernandes et al. [11] combined waste motor oil and recycled motor oil residues with polymers such as used polyethylene (PE), rubber powder, and styrene–butadiene–styrene block copolymer (SBS) to reduce the use of asphalt. The findings demonstrated that combining waste motor oil products with polymers enhanced the high-temperature characteristics. Lei et al. [12] prepared asphalt mastic using bio-oil and mineral powder filler with different dosages. The findings indicated that adding bio-oil enhanced asphalt mastic’s high-temperature stabilization and antideformation ability. Zheng et al. [10] used 4,4′-diphenylmethane diisocyanate (MDI) as an additive to boost the characteristics of wood-based bio-oil asphalt. The findings indicated that wood-based bio-oil asphalt will have strengthened high-temperature characteristics, and the low-temperature characteristics were not weakened. Ju et al. [13] used polyphosphoric-acid-modified castor oil bitumen, and the results showed that the polyphosphoric acid was not weakened. The wood bio-oil asphalt will raise high-temperature characteristics, and the low-temperature characteristics will not be weakened. Peng et al. [14] modified bioasphalt using rock asphalt. Zhao et al. [15] modified bioasphalt using nanomaterials, SBS, styrene–butadiene rubber (SBR), PE, and ethylene vinyl acetate (EVA). The high-temperature characteristics were remarkably enhanced when 7% bio-oil, 5% SBS, and 0.2% nanosilica were used.

Existing studies have found that the modifiers used to enhance bioasphalt performance are mainly organic modifiers. The most widely used are polymers such as SBS and SBR, which have excellent deformation and aging resistance under high-temperature conditions [16]. However, they also have the disadvantages of a cumbersome preparation process, high cost, and difficult storage. In addition to organic modifiers, inorganic modifiers could also effectively enhance the performance of asphalt. Commonly used inorganic modifiers include carbon black, fiber, diatomaceous earth, cement, slaked lime, silica fume, layered silicate, nano calcium carbonate, etc. Sun et al. [17] investigated the effect of carbon black (CB) on asphalt properties. It was found that CB enhances the properties of asphalt, such as its high-temperature characteristics and aging resistance. Zhu et al. [18] researched the effect of the type of layered silicate on the composite ratio of multidimensional nanomaterials (MDNs) in styrene–butadiene–styrene copolymer-modified asphalt (SBSMA). Li et al. [19] explored the effect of surface-modified diatomaceous earth (SMD) on the characteristics of bioasphalt, and the addition of SMD raised the asphalt’s characteristics, such as viscosity, softening point, fatigue index, and rutting coefficient. Yadykova et al. [20] modified bioasphalt using montmorillonite and found that montmorillonite significantly raises the characteristics of asphalt, such as elasticity, stiffness, and cohesion. Silica fume (SF), as an admixture for cement or concrete, has the advantages of great strength, abrasion resistance, erosion resistance, and corrosion resistance. Due to its peculiar micropore structure and better adsorption capacity, SF was often used as a modifier in preparing modified asphalt [21,22]. Yang et al. [23] used SF as an asphalt modifier. The findings indicated that SF could enhance asphalt’s high-temperature and temperature-affected characteristics. It could enhance the mixture’s high-temperature stabilization and water stability. Luo et al. [24] used SF to modify SBS asphalt, and the asphalt’s high temperature and water stability were significantly enhanced. Shang et al. [25] analyzed SF-modified asphalt’s rheological properties and microstructure before and after aging. Feng et al. [26] revealed that SF could enhance the high-temperature characteristics of asphalt. Yadykova et al. [27] examined the effect of hydrophilic and hydrophobic silica on bio-oil asphalt’s rheological properties, cohesion, and adhesion characteristics. Numerous studies have found that SF with a large specific surface area and strong adsorption capacity could usefully strengthen the high-temperature characteristics [28,29].

**Figure 1:**
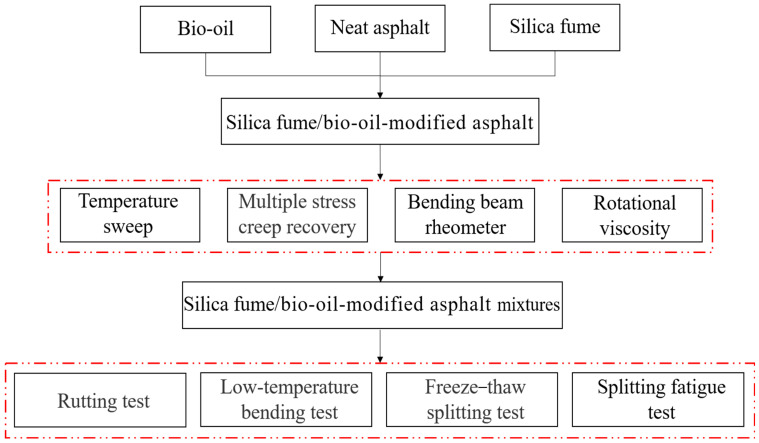
The experimental flowchart.

Therefore, in view of the current situation that bioasphalt’s high-temperature characteristics and antiaging are insufficient, SF was used as a modifier to modify bioasphalt in this study. The dosage of bio-oil in bioasphalt was 5%. The dosage of SF was 2–10% of the bioasphalt. A high-speed shear and colloid mill prepared the bio-oil/SF-modified asphalt (Bio + SF). The high- and low-temperature characteristics, temperature stabilization, and antiaging behavior of Bio + SF were investigated by rheological tests. At the same time, the road behavior of Bio + SF mixtures was tested. It provides the experimental basis and technological guidance for applying SF-modified bioasphalt. An experimental flowchart is illustrated in Figure 1.

## 2. Test Materials and Methods

### 2.1. Raw Materials

#### 2.1.1. Matrix Asphalt

This study selected the SK-70 model matrix asphalt as the original material. The detailed characteristics are outlined in Table 1.

#### 2.1.2. Silica Fume

Qinghai Tongda Company (Xining, China) produced the silica fume (SF) used in this study. The detailed characteristics are depicted in Table 2.

#### 2.1.3. Bio-Oil

In this study, the bio-oil used was from Jinan Boao Chemical Co., Ltd. (Jinan, China), and was the residue extracted from vegetable oil. The major components were 60–80% fatty acid and vegetable alcohol. The major features of the bio-oil are depicted in Table 3.

#### 2.1.4. Aggregates

In this study, the aggregate used was limestone. It was tested according to the JTG E42-2005 [35] specification, and all the indexes satisfied the technical demands of the standard. The specific technical indexes of coarse and fine aggregates are depicted in Table 4 and Table 5.

#### 2.1.5. Grading Curve

**Figure 2:**
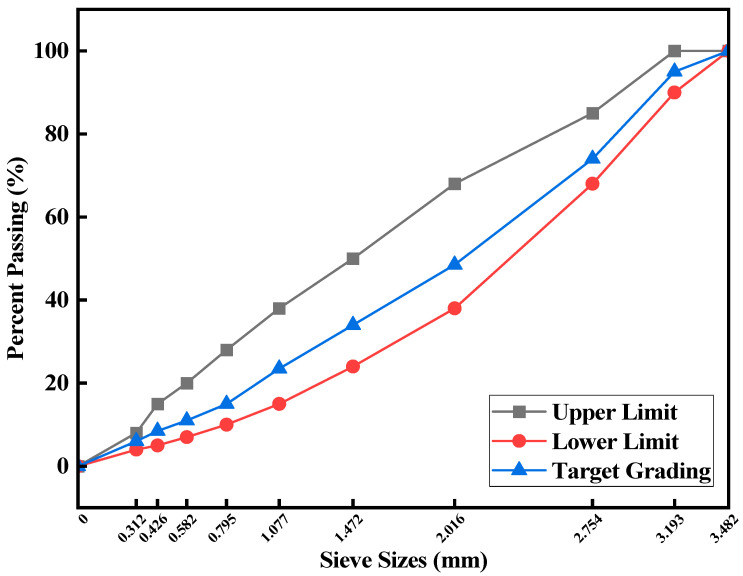
Grading curve.

AC-13 grading was selected for this study, and the grading curve is displayed in Figure 2.

### 2.2. Preparation of Bio-Oil/SF-Modified Asphalt

Since SF is a chainlike aggregate from amorphous spheres, it is easily adsorbed to moisture in the air due to its large particle size and large specific surface area. To ensure the SF remained dry during the preparation process, the SF was put into an oven at 105 °C to dry before preparing the modified asphalt. The degree of dispersion of the SF in the asphalt directly affects the performance of modified asphalt. To minimize the phenomenon of SF agglomeration in asphalt, in the preparation of SF/bio-oil-modified asphalt (Bio + SF), an MJ-65 colloid mill was used for dispersion for 3 min at a speed of 2900 r/min. The preparation process of Bio + SF was as follows: First, 400 g of matrix asphalt was put into an oven at 135 °C for 1 h to reach the flow state; then 5% (by mass of asphalt) of bio-oil was incorporated. After premixing using a glass rod, the mixture was sheared at 135 °C and 3000 rpm for 5 min. After shearing, at 180 °C, the predried SF was added to the bioasphalt, dispersed for 3 min using a colloid mill, and mixed at 5000 rpm for 30 min using a high-speed shear. Finally, Bio + SF was obtained. The dosage of SF was 0%, 2%, 4%, 6%, 8%, and 10% of the mass of bioasphalt, respectively. Abbreviations are presented in Table 6.

### 2.3. Test Methods

#### 2.3.1. Temperature Sweep (TS)

Dynamic shear rheometers were used to perform temperature sweeps according to the AASHTO T 315-20 [36] specification. The temperature range was 52–82 °C, with temperature increments of 6 °C. The test was conducted under stress-controlled mode. The sample diameter was 25 mm, and the thickness was 1 mm. The frequency was 10 rad/s. The original asphalt strain was set to 12%, and the strain after rolling thin-film oven (RTFO) aging was set to 10%.

#### 2.3.2. Multiple Stress Creep Recovery (MSCR)

The MSCR test on asphalt after RTFO was performed according to the AASHTO T350-14 [37] specification. The MSCR test consisted of 0.1 kPa and 3.2 kPa stress levels. A total of 20 cycles were loaded at 0.1 kPa stress, and 10 cycles were loaded at 3.2 kPa stress. The temperature was 58 °C. Three parallel tests were run for each type of asphalt.

#### 2.3.3. Rotational Viscosity

Based on the AASHTO T 316 [38] specification, a Brookfield viscometer performed viscosity tests on unaged asphalt. The test was conducted using a No. 27 rotor. The rotor and sample container were maintained in an oven for 1.5 h. The samples were loaded into a viscometer, held to reach the test equilibrium temperature, and then tested. The torque was maintained between 10% and 98% during the test. The test temperatures were 135 °C, 165 °C, and 175 °C. The rotational speed was 20 r/min at 135 °C. The rotational speed was 50 r/min at 165 °C and 175 °C. After the viscosity value was stabilized, readings were taken at 60 s intervals for 3 consecutive times, and the average value was used as the viscosity measurement value. Each asphalt was tested twice in parallel.

#### 2.3.4. Bending Beam Rheometer (BBR)

The low-temperature rheological characteristics of Bio + SF were tested according to AASHTO T 313-19 [39]. The size of the specimen was 127 mm × 6.35 mm × 12.7 mm. The contact load was 35 ± 10 mN. The test load was 980 ± 50 mN. The test load was constant for 240 s. The temperatures were −12 °C and −18 °C. The strength modulus (S) and creep rate (m) indices for PAV-aged asphalt were obtained. Each asphalt was tested three times in parallel.

### 2.4. Asphalt Mixture Road Behavior Test

According to the JTG E20-2011 [40] specification, the appropriate mixing and compaction temperatures were selected according to the Brookfield rotational viscosity test. The optimum asphalt–aggregate ratio was obtained using the Marshall test method.

#### 2.4.1. Rutting Resistance at High Temperature

According to the specification of JTG E20-2011 [40], the rutting test was carried out on matrix asphalt mixtures, 5%Bio + 6%SF mixtures, 5%Bio + 8%SF mixtures, and 5%Bio + 10%SF mixtures. The specimen size was 300 mm × 300 mm × 50 mm. The temperature was 60 °C. The contact pressure between the tire and the specimen was 0.7 MPa, and the wheel acted on the specimen 42 times per minute. The rutting deformations d_1_ and d_2_ at 45 min (t_1_) and 60 min (t_2_) were taken for the calculation of dynamic stability (DS), as shown in Equation (1).

(1) $$DS=\frac{42\times (t_{2}-t_{1})}{d_{2}-d_{1}}$$

#### 2.4.2. Low-Temperature Anticracking Properties

Based on the specification of JTG E20-2011 [40], a low-temperature bending beam damage test was carried out for matrix asphalt mixtures, 5%Bio + 6%SF mixtures, 5%Bio + 8%SF mixtures, and 5%Bio + 10%SF mixtures. The specimen size was 250 mm × 30 mm × 35 mm, a small beam specimen. The loading rate was 50 mm/min. The temperature was −10 °C. The ultimate bending tensile failure strain (ε) was calculated as in Equation (2):

(2) $$\varepsilon =\frac{6hd}{L^{2}}$$

where L is the diameter of the specimen, mm; h is the height of the midspan section of the specimen, mm; and d is the midspan deflection of the specimen, mm.

#### 2.4.3. Freeze–Thaw Splitting Strength

According to the specification of JTG E20-2011 [40], freeze–thaw splitting tests were conducted on matrix asphalt mixtures, 5%Bio + 6%SF mixtures, 5%Bio + 8%SF mixtures, and 5%Bio + 10%SF mixtures. The specimens of each asphalt mixture were divided into two groups. One group was first treated with a freeze–thaw cycle, and then the two groups of specimens were soaked in a 25 °C water bath for 2 h and then tested for splitting strength separately. The freeze–thaw splitting strength ratio (TSR) formula is presented in (3):

(3) $$TSR=\frac{R_{T2}}{R_{T1}}\times 100\%$$

where R_T1_ and R_T2_ are the strength values of the asphalt mixture before and after freezing and thawing; R = 0.006287 P/h, MPa; P is the breaking load, N; and h is the height of the specimen, mm.

#### 2.4.4. Splitting Fatigue

The durability test was tested using the split fatigue test on matrix asphalt mixtures, 5%Bio + 6%SF mixtures, 5%Bio + 8%SF mixtures, and 5%Bio + 10%SF mixtures. The temperature was 15 °C. The stress level was 0.3 MPa. Control mode was selected as stress control. The loading frequency was 10 Hz.

## 3. Results and Analysis

### 3.1. TS Analysis

**Figure 3:**
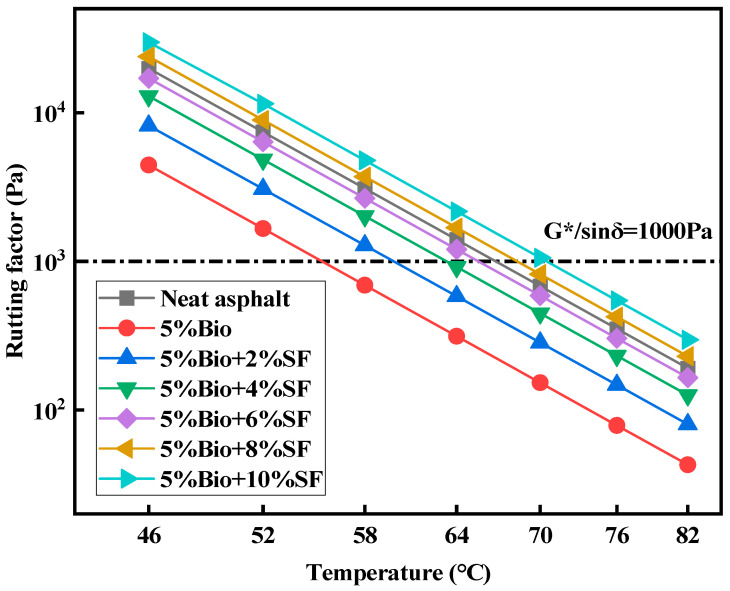
Rutting factor of unaged asphalt.

**Figure 4:**
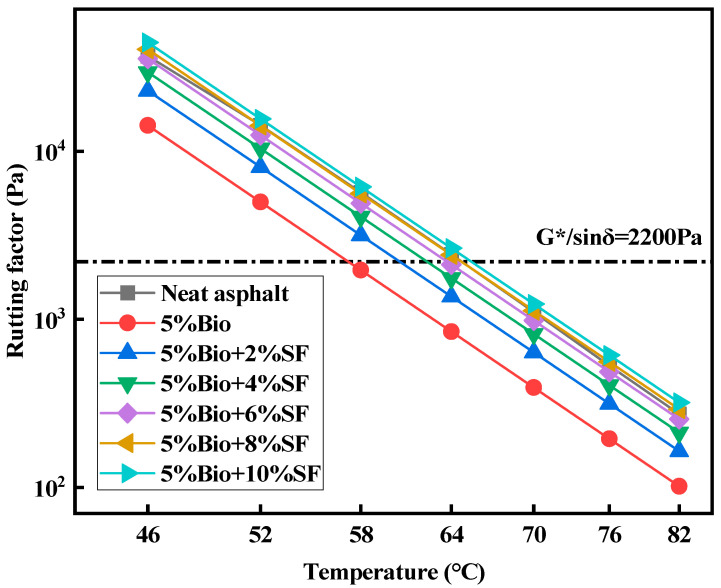
Rutting factors for short-term aged asphalt.

The rutting factor (G*/sinδ) is utilized to assess the ability of asphalt to resist irrecoverable deformation during dynamic shear. The larger its value, the more superior the capacity of asphalt to resist deformation [41,42]. Figure 3 and Figure 4 show the rutting factor with temperature curves of matrix asphalt, bioasphalt, and bio-oil/SF with different SF dosages after unaged and short-term aging, respectively.

G*/sinδ decreased with increasing temperature for varying types of asphalt (Figure 3 and Figure 4). Adding bio-oil reduced the G*/sinδ value of asphalt compared with matrix asphalt. The G*/sinδ value of bioasphalt declined by 77.6% compared with matrix bitumen at 52 °C, which substantially weakened the antideformation characteristics of the matrix asphalt. This was attributed to the fact that adding bio-oil increases the asphalt’s lightweight component and reduces the asphalt’s ability to resist high-temperature deformation [14]. The G*/sinδ of bioasphalt strengthened continuously after incorporating SF into bioasphalt. The G*/sinδ values of bio-oil + SF strengthened by 85.3%, 191.9%, 284.3%, 437.0%, and 591.8% for 2%SF, 4%SF, 6%SF, 8%SF, and 10%SF dosages at 52 °C, respectively. Adding SF enhanced the ability of bioasphalt to prevent permanent distortion. When the amount of SF was 6%, the G*/sinδ value was comparable with matrix asphalt. When the amount of SF was 8%, the antirutting characteristics of bioasphalt at high temperatures were superior to those of matrix asphalt.

According to the Superpave standard, G*/sinδ is an index of the PG gradation of asphalt at high temperatures. The standard defines that the G*/sinδ of unaged asphalt shall be greater than 1000 Pa. The G*/sinδ of asphalt after short-term aging shall be greater than 2200 kPa. The results of PG grading are depicted in Table 7.

According to the asphalt high-temperature PG results shown in Table 7, SF can effectively promote the capacity of bioasphalt to withstand permanent deformation and enhance its high-temperature stabilization.

### 3.2. Antiaging Performance

**Figure 5:**
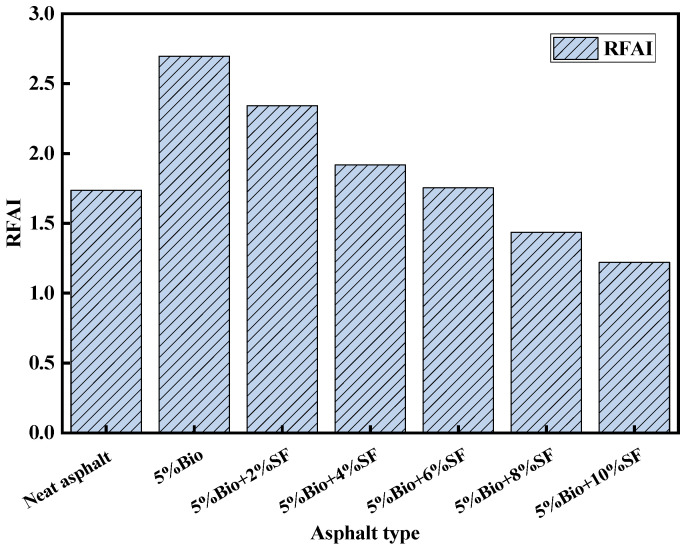
RFAI values for different asphalt types.

The rutting factor aging index (RFAI) was calculated based on the rutting factor test results of original and short-term aged asphalt in the temperature sweep. RFAI is calculated as shown in Equation (4). The lower the RFAI, the more superior the antiaging characteristics [43,44]. The results of RFAI values for different asphalts are shown in Figure 5.

(4) $$RFAI=\frac{Rutting\;factor/short-term\;aged}{Rutting\;factor/unaged}$$

As illustrated in Figure 5, The RFAI value of bio-asphalt appeared to be increased, with the RFAI elevated by 55.2% compared with the matrix asphalt. Meanwhile, the RFAI value of bio-asphalt continuously decreases after SF incorporation. Compared with 5%Bio, the RFAI values of 5%Bio + 2%SF, 5%Bio + 4%SF, 5%Bio + 6%SF, 5%Bio + 8%SF and 5%Bio + 10%SF decreased by 13.1%, 28.9%, 35.0%, 46.8% and 54.7%, respectively. The resistance to aging of bio-asphalt strengthened with the rise of the SF content. When the SF amount was 8%, bio-asphalt’s anti-aging characteristics were better than matrix asphalt. The cause was mainly related to the composition of bio-oil. Bio-oil contains many light components, which easily volatilize in a high-temperature oxygen-containing environment and are gradually converted into heavy components, weakening bio-asphalt’s resistance to aging characteristics. Incorporating SF could effectively enhance the force between SF particles and asphalt molecules, creating a stable space network structure and greatly enhancing the cohesion of bio-based materials and matrix asphalt. Then, it strengthened the resistance to aging characteristics of bio-asphalt.

### 3.3. MSCR Analysis

**Figure 6:**
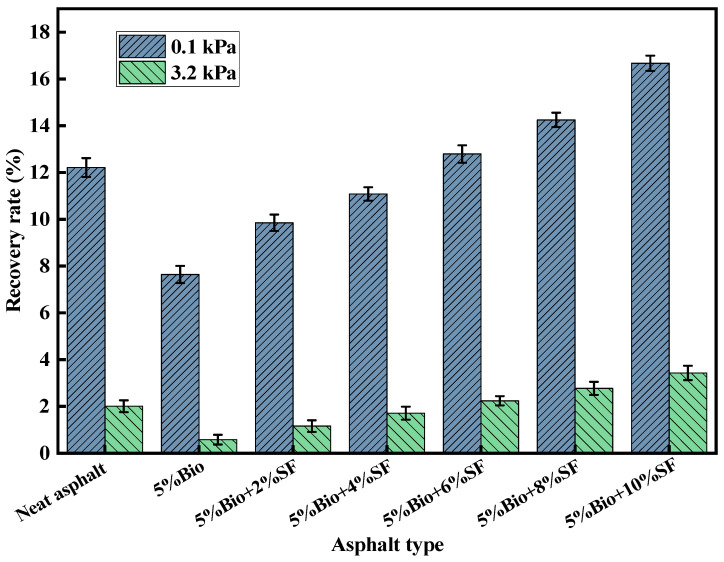
Creep recovery for different asphalt types.

**Figure 7:**
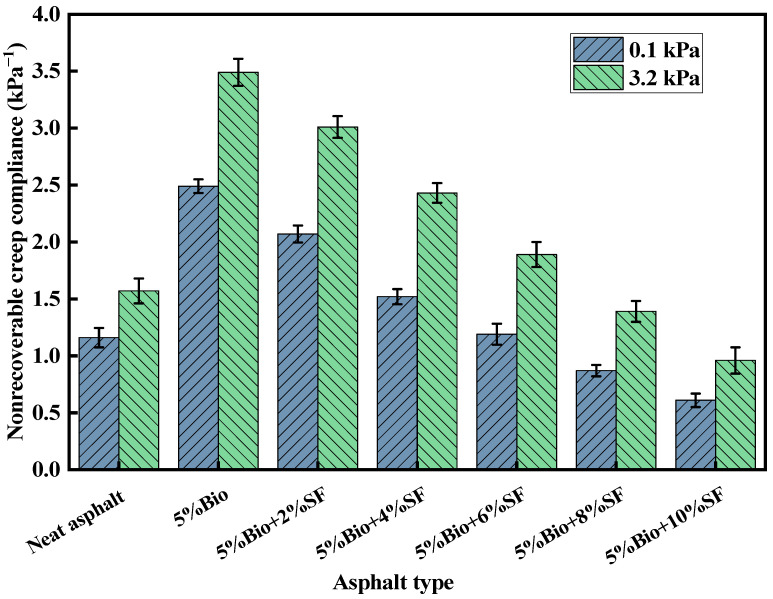
Unrecoverable creep flexibility for different asphalt types.

The creep recovery rate (R) and the unrecoverable creep flexibility (Jnr) could be obtained from the MSCR test. The R-value characterizes the resilient composition. The higher the value, the greater the elastic deformation recovery. The Jnr-value characterizes the resistance to permanent deterioration. The lower the value, the greater the antideformation ability at high-temperature terms [16,41]. Figure 6 and Figure 7 display the R-values and Jnr-values of asphalt for stress levels of 0.1 kPa and 3.2 kPa at 58 °C, respectively.

The R-values of varying asphalt types decline continuously with rising stress (Figure 6). It indicates that an increase in stress level decreases the capacity of asphalt to restore deformation under a high-temperature environment resiliently. The R-value of bioasphalt declined compared with matrix asphalt at the corresponding stress level. The R-value of bioasphalt declined by 37.4% at 0.1 kPa. This was mainly because the bio-oil contained more lightweight components, which reduced the resilient composition of the asphalt, thus reducing the deformation capacity of the asphalt for elastic recovery. SF incorporation strengthened the deformation recovery capacity of bioasphalt under high-temperature conditions. At 0.1 kPa, compared with 5%Bio-oil, the R-values of 5%Bio + 2%SF, 5%Bio + 4%SF, 5%Bio + 6%SF, 5%Bio + 8%SF, and 5%Bio + 10%SF increased by 28.9%, 45.0%, 67.4%, 86.5%, and 118.2%, respectively. When the amount of SF was 6%, the R-value of Bio + SF was similar to that of matrix asphalt. When the amount of SF was more than 6%, the R-value of Bio + SF was significantly larger than that of matrix asphalt, and the deformation capacity of elastic recovery was better than that of matrix asphalt.

The Jnr-values of varying asphalt types increased with increasing stress (Figure 7). The higher the stress level, the bigger the irrecoverable deformation of asphalt under repeated loading. The Jnr-value of bioasphalt was significantly higher than that of matrix asphalt at 0.1 kPa, and the Jnr-value rose by 114.7%. After the incorporation of SF, the Jnr-values of bioasphalt decreased continuously. Compared with that of 5%Bio, the Jnr-values of 5%Bio + 2%SF, 5%Bio + 4%SF, 5%Bio + 6%SF, 5%Bio + 8%SF, and 5%Bio + 10%SF were reduced by 16.9%, 39.0%, 52.2%, 65.1%, and 75.5%, respectively. At 3.2 kPa, the Jnr-value of 5%Bio + 6%SF was similar to that of matrix asphalt. When the dosage of SF was greater than 6%, the Jnr-value of Bio + SF was significantly smaller than that of matrix asphalt, and the resistance to deformation was superior to that of matrix asphalt.

### 3.4. Viscosity Results Analysis

**Figure 8:**
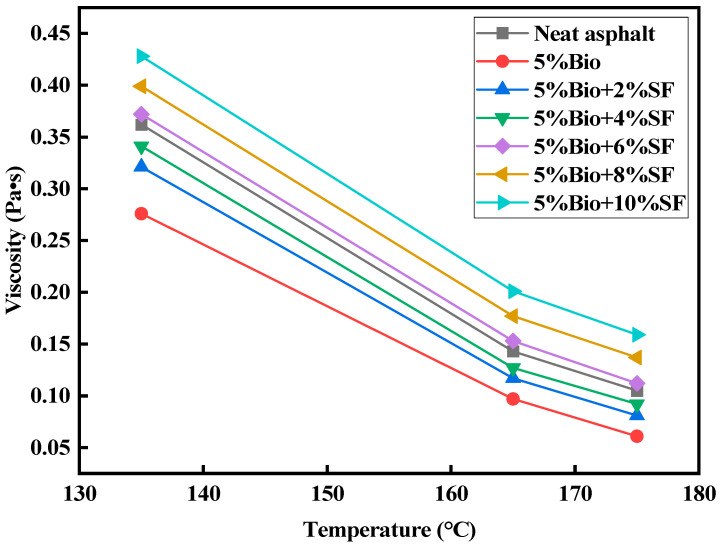
Viscosity test results.

The viscosity of asphalt is an index for evaluating the ease of pavement construction. Figure 8 displays the viscosity values of varying asphalt types at 135 °C, 165 °C, and 175 °C.

The viscosity values of varying asphalt types declined with increasing temperature (Figure 8). The viscosity of 5%Bio was 0.276 Pa·s at a temperature of 135 °C. The viscosity of bioasphalt declined by 23.8% compared with that of matrix asphalt. After adding SF, bioasphalt viscosity gradually increased, and the deformation resistance was enhanced. Compared with that of 5%Bio, the viscosity values of 5%Bio + 2%SF, 5%Bio + 4%SF, 5%Bio + 6%SF, 5%Bio + 8%SF, and 5%Bio + 10%SF were increased by 16.3%, 23.6%, 34.8%, 44.6%, and 55.1%, respectively. When the SF dosage was 6%, the viscosity of Bio + SF was comparable with the matrix asphalt.

Asphalt binders are susceptible to temperature, and temperature sensitivity is an essential characteristic. Based on the viscosity data of asphalt at 135 °C, 165 °C, and 175 °C, regression analyses were performed for varying asphalt types according to the viscosity–temperature curve equation. The formula is shown in Equation (5) [43].

(5) $$\log(\log\left(\eta \times 10^{3}\right))=n-m\mathrm{lg}\left(T+273.13\right)$$

**Figure 9:**
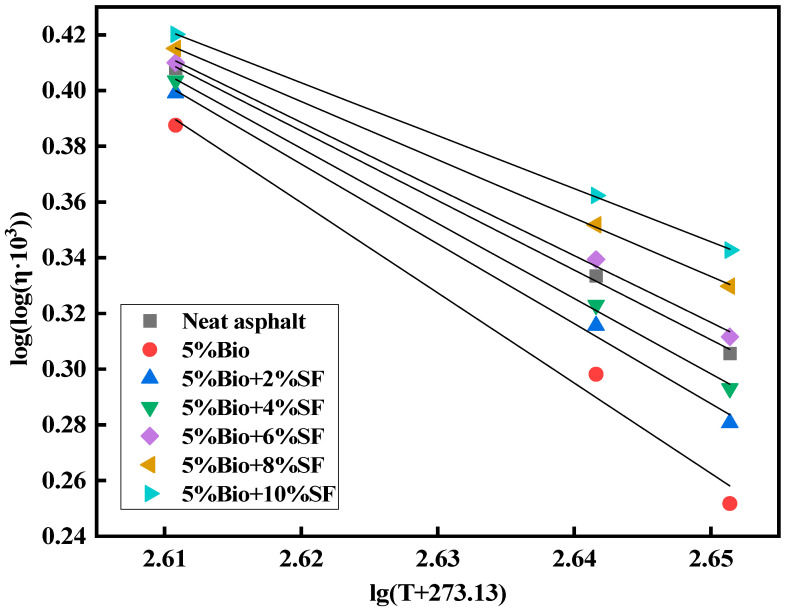
Viscosity–temperature curve.

In the equation, m is the viscosity–temperature indices VTS. The higher the value, the bigger the characterization of the asphalt material viscosity by temperature and the worse its temperature stability. Figure 9 displays the viscosity–temperature curves of varying asphalts, and the fitting parameters are summarized in Table 8.

Adding bio-oil diminished the viscosity of asphalt (Figure 9 and Table 8). Adding bio-oil increased the VTS value by 22.9% compared with matrix asphalt, weakening the temperature stability. After adding SF, the VTS value of bioasphalt was reduced with the increase in SF amount, which enhanced temperature stability. The viscosity values of 5%Bio + 2%SF, 5%Bio + 4%SF, 5%Bio + 6%SF, 5%Bio + 8%SF, and 5%Bio + 10%SF decreased by 11.5%, 16.8%, 26.2%, 35.4%, and 41.3%, respectively, compared with 5%Bio. When the SF dosage was 6%, bioasphalt’s VTS values were comparable with matrix asphalt’s. This was mainly because SF had a large specific area of surface, which enhanced the force of interaction with the asphalt and bio-oil molecules. This led to the formation of a stable space network structure in the asphalt, which enhanced bioasphalt’s temperature stabilization.

### 3.5. Low-Temperature Rheological Characteristics

**Figure 10:**
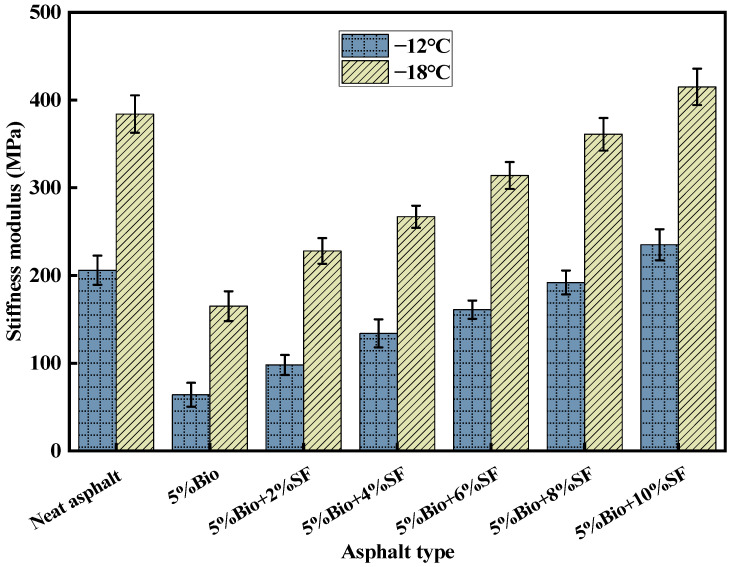
S-values of varying asphalt types.

**Figure 11:**
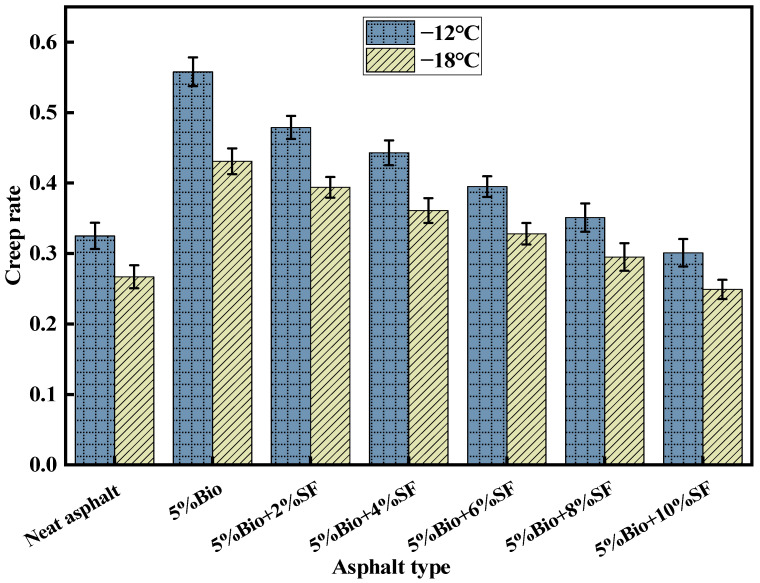
m-Values for varying asphalt types.

The S characterizes the resistance of asphalt to loads. The m characterizes the change rate of asphalt stiffness with time. Based on the AASHTO T 313-19 specification, the S-value shall be less than 300 MPa, and the m-value shall be bigger than 0.3 [45,46,47]. Figure 10 and Figure 11 demonstrate the test results of varying asphalt types.

The S-values of varying asphalt types increased with decreasing temperature (Figure 10). The lower the temperature, the lower the capacity of asphalt to resist loading. At −12 °C, the S-value of varying asphalt types was less than 300 MPa, which could satisfy the standard request. When the temperature was −18 °C, only the S-values of 5%Bio, 5%Bio + 2%SF, and 5%Bio + 4%SF asphalts could satisfy the standard demand. The S-values of matrix asphalt, 5%Bio + 6%SF, 5%Bio + 8%SF, and 5%Bio + 10%SF were all greater than 300 MPa, and the ability to resist low-temperature loading was significantly reduced. Among them, when the temperature was −18 °C, adding bio-oil minimized the S-value of matrix asphalt with a decrease of 57.0%. The low-temperature antifracture characteristics of bioasphalt were significantly enhanced. This was because bio-oil could supplement the lightweight component and reduce the creep modulus during the aging process of asphalt. When SF was incorporated, compared with 5%Bio, the S-values of 5%Bio + 2%SF, 5%Bio + 4%SF, 5%Bio + 6%SF, 5%Bio + 8%SF, and 5%Bio + 10%SF increased by 38.2%, 61.8%, 90.3%, 118.8%, and 151.5%, respectively. The incorporation of SF diminished the low-temperature behavior of bioasphalt. The S-value of bioasphalt was comparable with that of matrix asphalt when the amount of SF was 8%. This was because the intermolecular interaction between bio-oil, SF, and asphalt was enhanced, and the structure was stabilized after the incorporation of SF. It increased asphalt’s modulus, which caused it to crack easily under low-temperature conditions.

The m-values of asphalt decreased with decreasing temperature (Figure 11). This demonstrates that the stress relaxation ability of asphalt diminished when the temperature was reduced, leading to easy cracking of asphalt. The m-value of all seven asphalts was greater than 0.3 at −12 °C, which met the specification. The m-value of matrix asphalt was 0.267 at −18 °C, which did not satisfy the standard demand. The m-value of asphalt strengthened when bio-oil was blended into the matrix asphalt and increased by 61.4% compared with the m-value of matrix asphalt. The m-value of bioasphalt declined markedly after incorporating different amounts of SF into the bioasphalt. Compared with 5%Bio, the m-values of 5%Bio + 2%SF, 5%Bio + 4%SF, 5%Bio + 6%SF, 5%Bio + 8%SF, and 5%Bio + 10%SF showed a decrease of 8.6%, 16.2%, 23.9% 31.6%, and 42.2%, respectively. The m-value of Bio + SF was comparable with that of matrix asphalt when the SF dosage was 8%.

**Figure 12:**
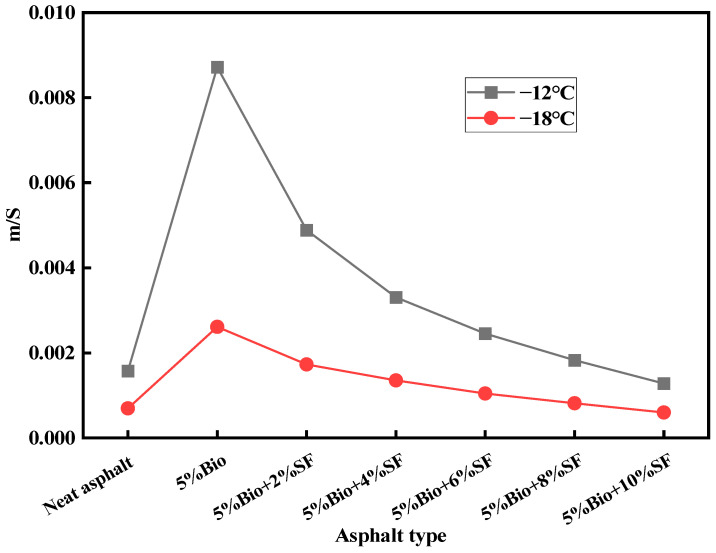
m/S values of varying asphalt types.

To better estimate the low-temperature anticracking behavior of Bio + SF, the creep recovery rate (m)/strength modulus (S) index was used for comparative analysis. Among them, the greater the m/S is, the more superior the anticracking characteristics of asphalt are, and vice versa [48]. Figure 12 shows the variation pattern of m/S values for asphalt at different temperatures.

As illustrated in Figure 12, the lower the temperature, the higher the m/S value of asphalt. The m/S value of bioasphalt strengthened by 452.6% compared with that of matrix asphalt at −12 °C. Incorporating bio-oil strengthened the anticracking characteristics of matrix asphalt. The m/S value of bioasphalt decreased after SF was incorporated into the bio-asphalt. Compared with 5%Bio, the m/S values of 5%Bio + 2%SF, 5%Bio + 4%SF, 5%Bio + 6%SF, 5%Bio + 8%SF, and 5%Bio + 10%SF decreased by 43.9%, 62.1%, 71.9%, 79.0%, and 85.3%, respectively. The m/S values were ranked as follows: 5%Bio > 5%Bio + 2%SF > 5%Bio + 4%SF > 5%Bio + 6%SF > 5%Bio + 8%SF > matrix asphalt > 5%Bio + 10%SF. SF incorporation weakened the anticracking behavior of bioasphalt. However, for a certain amount of incorporation, the low-temperature behavior of Bio + SF was still better than that of matrix asphalt.

Based on comprehensive G*/sinδ, R, Jnr, RFAI, VTS, m, S, m/S indexes, adding bio-oil reduced the high-temperature stabilization of asphalt, but bioasphalt had superior low-temperature behavior. The incorporation of SF could effectively compensate for the high-temperature antideformation characteristics of bioasphalt and enhance the aging resistance and temperature stability. However, SF will reduce the low-temperature characteristics of bioasphalt. Nevertheless, with a certain amount of SF dosage, the low-temperature behavior of bioasphalt was still superior to matrix asphalt’s.

### 3.6. Road Performance

#### 3.6.1. Rutting Resistance at High Temperature

**Figure 13:**
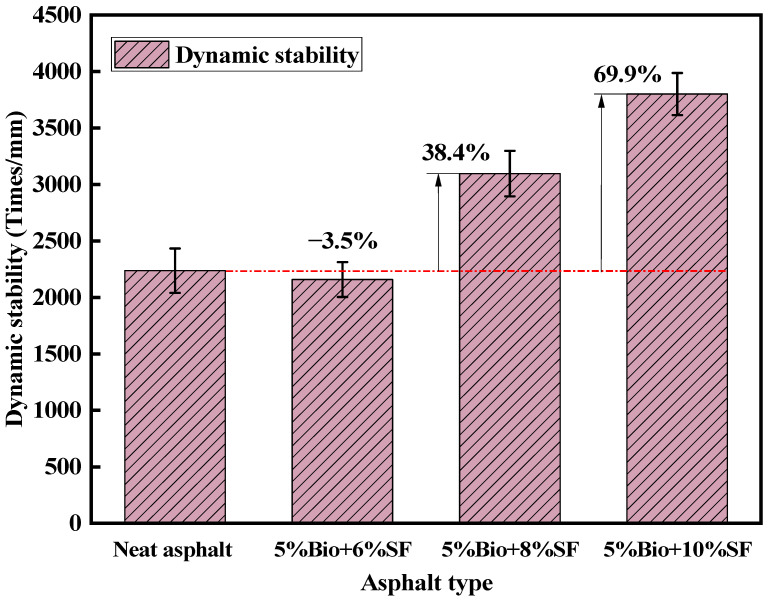
Dynamic stability test results.

The four asphalt mixtures’ dynamic stability (DS) was obtained by high-temperature rutting tests, as shown in Figure 13.

According to JTG D50-2006 [49], the DS of an ordinary asphalt mixture should be superior to 1000 times/mm in the hot summer area. The DS of the modified asphalt mixture should be superior to 2800 times/mm. The DS of the matrix asphalt mixture was 2237 events/mm (Figure 13). Compared with the matrix asphalt mixtures, the 5%Bio + 6%SF mixture showed a decrease of 3.5% in dynamic stability. The 5%Bio + 8%SF and 5%Bio + 10%SF mixtures increased by 38.4% and 69.9%, respectively. The DS of bio-oil + SF mixtures with more than 8%SF was more than 2800 times/mm, which met the standard demand and was excellent for the matrix asphalt mixtures. Incorporating SF could effectively strengthen the antirutting characteristics of bioasphalt and asphalt mixtures.

#### 3.6.2. Low-Temperature Anticracking Characteristics

**Figure 14:**
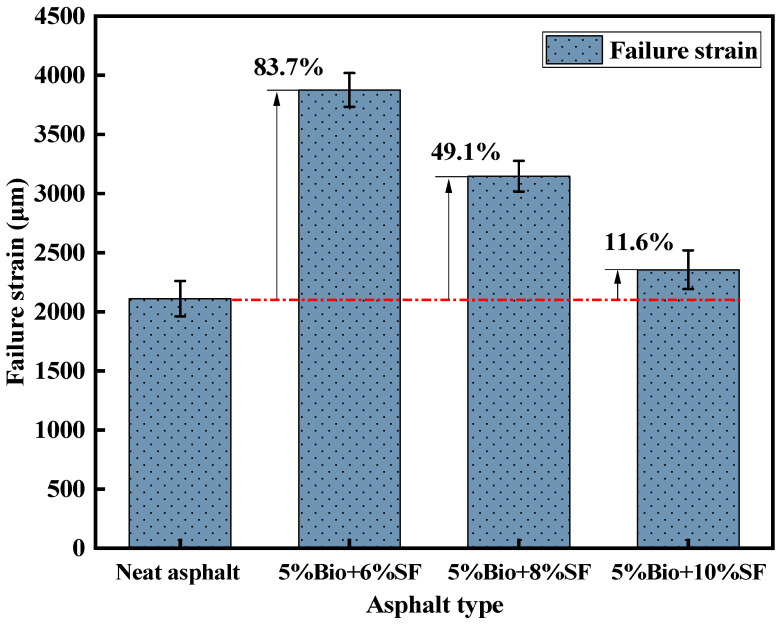
Failure strain test results.

The destructive strain of asphalt mixture specimens under a low-temperature environment is to reflect the capacity of asphalt mixture deformation. The stronger the value, the more superior the capacity of the mixture to deform. Figure 14 displays the results of the destructive strain tests of four asphalt mixtures.

According to JTG D50-2006 [49], the failure strain of ordinary asphalt mixtures is not less than 2000 μm. Modified asphalt mixtures are not smaller than 2500 μm. As illustrated in Figure 14, the failure strain of matrix asphalt mixtures is 2111 μm, which can satisfy the standard demand. After adding bio-oil and SF to the matrix asphalt, the failure strain of the bio-oil + SF mixtures strengthened remarkably, and the deformation resistance of the mixtures was effectively improved. Compared with the matrix asphalt mixtures, the failure strains of 5%Bio + 6%SF, 5%Bio + 8%SF, and 5%Bio + 10%SF mixtures increased by 83.7%, 49.1%, and 11.6%, respectively. The failure strain of 5%Bio + 10%SF modified asphalt with a 10%SF dosage was 2356 μm, which did not satisfy the standard demand for the failure strain of modified asphalt mixtures. The reason was that bio-oil contains more lightweight components, which could effectively reduce the creep modulus of asphalt. Thus, the low-temperature anticracking behavior of bio-oil + SF mixtures can be enhanced. However, incorporating SF enhanced the intermolecular forces, increasing the modulus of bioasphalt, resulting in bioasphalt mixtures being prone to cracking under low-temperature environments. The higher the amount of SF, the poorer the low-temperature behavior of bioasphalt mixtures.

#### 3.6.3. Water Damage Resistance

**Figure 15:**
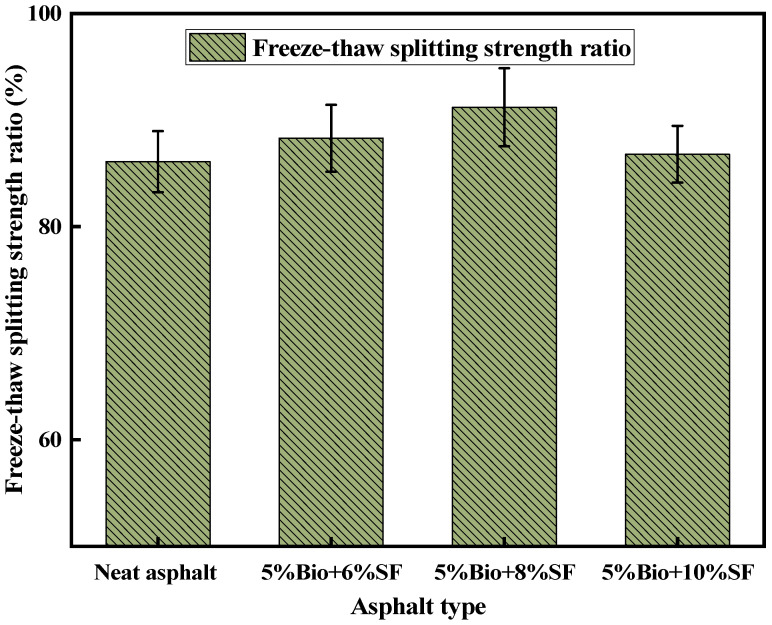
Freeze–thaw splitting strength ratio test results.

The freeze–thaw splitting test is an index to assess the water loss resistance of asphalt mixtures. Figure 15 exhibits the results of the freeze–thaw split strength ratio (TSR) of four asphalt mixtures: matrix asphalt, 5%Bio + 6%SF, 5%Bio + 8%SF, and 5%Bio + 10%SF.

According to JTG D50-2006 [49], the TSR of ordinary asphalt mixtures is not smaller than 75%. The modified asphalt mixture is not smaller than 80%. As shown in Figure 15, the TSR of the matrix asphalt mixture was 86.1%, which satisfies the standard demand. The TSRs of 5%Bio + 6%SF, 5%Bio + 8%SF, and 5%Bio + 10%SF mixtures were increased by 2.6%, 5.9%, and 0.8% compared with that of the matrix asphalt mixture, respectively, which satisfy the standard demand. This was because incorporating SF can enhance the adhesion between asphalt and mineral material, enhancing the water damage resistance of bio-oil + SF mixtures.

#### 3.6.4. Fatigue Life

**Figure 16:**
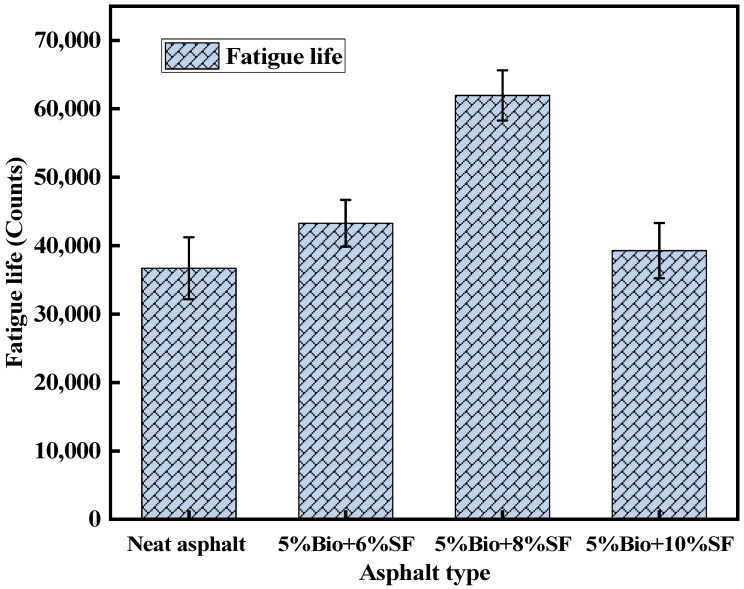
Fatigue life test results.

Fatigue life is an essential parameter for evaluating the durability of asphalt mixtures. Figure 16 exhibits the fatigue life test results of four asphalt mixtures for matrix asphalt, 5%Bio + 6%SF, 5%Bio + 8%SF, and 5%Bio + 10%SF.

As demonstrated in Figure 16, the fatigue life of bio-oil + SF mixtures was greater than the matrix asphalt mixtures at the 0.3 MPa stress level. Compared to the fatigue life of the matrix asphalt mixtures, the fatigue life of 5%Bio + 6%SF, 5%Bio + 8%SF and 5%Bio + 10%SF mixtures increased by 17.9%, 68.9% and 7.0%, respectively. The incorporation of SF resulted in a remarkably enhanced fatigue life of bio-asphalt.

## 4. Conclusions

This study developed bio-oil + SF, and TS, MSCR, viscosity, and BBR tests investigated the high- and low-temperature characteristics of bio-oil + SF. Meanwhile, the road behavior of bio-oil + SF mixtures was examined using a high-temperature stabilization test, flexural creep test, split freeze–thaw test, and fatigue test. The main conclusions were drawn:

(1) Incorporating bio-oil strengthened the low-temperature characteristics of matrix asphalt. However, bio-oil significantly reduced the high-temperature characteristics of matrix asphalt.
(2) The G*/sinδ, R, and Jnr indexes showed that adding SF could improve resistance to the permanent deformation of bioasphalt at high temperatures. The RFAI and VTS indexes showed that adding SF improved bioasphalt’s aging resistance and temperature stability.
(3) The m, S, and m/S indexes at −12 °C and −18 °C indicated that the rise in the dosage of SF weakened the low-temperature characteristics of bioasphalt. However, when the dosage of SF was less than 8%, the low-temperature characteristics of bio-oil + SF were still superior to those of matrix asphalt.
(4) The high-temperature stabilization, low-temperature anticracking, water damage resistance, and fatigue durability of bio-oil + SF mixtures were superior to those of matrix asphalt mixtures. The best road behavior was shown by 5%Bio + 8%SF mixtures.
(5) The test results of SF-modified bioasphalt and mixtures provided a specific scientific basis for the green and efficient application of bio-oil and SF. However, this study lacks an analysis of the SF + bio-oil mechanism. The next step will continue to explore the micromechanism of SF-modified bioasphalt and its practical engineering applications.

## Figures and Tables

**Table 1:** The detailed characteristics of asphalt.

Technical Indexes	Unit	Test Results	Specification Requirements	Test Methods
Penetration (25 °C, 5 s, 100 g)	0.1 mm	67	60~80	ASTM D 5 [30]
Ductility (5 cm/min, 10 °C)	cm	32	≥15	ASTM D 113 [31]
Softening point	°C	48	≥46	ASTM D 36 [32]
Viscosity (60 °C)	Pa·s	219	≥180	ASTM D 4402 [33]
after RTFOT aging				
Mass loss	%	0.024	≤±0.8	ASTM D 2872 [34]
Residual penetration ratio (25 °C, 5 s, 100 g)	%	67	≥61	ASTM D 5
Ductility (5 cm/min, 10°C)	cm	8	≥6	ASTM D 113

**Table 2:** SF technical indexes.

Indexes	Results
Appearance	Pale grayish-white powder
SiO_2_ content (%)	92.88
PH value	6–8
Whiteness (%)	0.24
Particle size distribution (μm)	0.1–0.3
Specific surface area (m^2^/g)	25.37

Notes: The whiteness of SF is defined as its brightness or lightness compared with a standard white reference material.

**Table 3:** The major features of bio-oil.

Indicators	Results
Acid value (mgKOH/g)	51
Moisture content (%)	≤0.3
Kinematic viscosity (mm^2^/s)	148
Density (g/mL)	0.93
Fatty acids content (%)	65

**Table 4:** Technical specifications of coarse aggregates.

Technical Indicators	Results	Standards
Los Angeles abrasion test (%)	23	≤28
Crushing value (%)	12.1	≤28
Acicular and flaky grain in aggregate (%)	10.3	≤15

**Table 5:** Technical specifications for fine aggregates.

Technical Indicators	Results	Standards
Water absorption (%)	0.5	≤2.0
Mud content (%)	1.7	≤3
Sand equivalent (%)	69	≥60

**Table 6:** Bio + SF abbreviations.

SF Content	0%	2%	4%	6%	8%	10%
Abbreviation	5%Bio	5%Bio + 2%SF	5%Bio + 4%SF	5%Bio + 6%SF	5%Bio+ 8%SF	5%Bio + 10%SF

**Table 7:** Asphalt high-temperature PG classification.

Type of Asphalt	High-Temperature Grade
Matrix asphalt	PG64
5%Bio	PG52
5%Bio + 2%SF	PG58
5%Bio + 4%SF	PG58
5%Bio + 6%SF	PG58
5%Bio + 8%SF	PG64
5%Bio + 10%SF	PG64

**Table 8:** Viscosity–temperature curve fitting parameters.

Type of Asphalt	*m*	*n*	R^2^
Matrix asphalt	2.497	6.928	0.99
5%Bio	3.239	8.845	0.98
5%Bio + 2%SF	2.866	7.883	0.99
5%Bio + 4%SF	2.696	7.443	0.99
5%Bio + 6%SF	2.392	6.657	0.99
5%Bio + 8%SF	2.092	5.876	0.99
5%Bio + 10%SF	1.901	5.384	0.99

## Data Availability

Some or all data, models, or codes generated or used during the study are available from the corresponding author by request.
